# Coping Strategies During the COVID-19 Pandemic and Self-Determination: A Review of Russian Studies

**DOI:** 10.11621/pir.2023.0201

**Published:** 2023-06-15

**Authors:** E.A. Kuznetsova, N.L. Moskvicheva, E.V. Zinovyeva, S.N. Kostromina

**Affiliations:** Saint Petersburg State University, Russia

**Keywords:** COVID-19, pandemic, stress, coping strategies, self-determination

## Abstract

**Background:**

The COVID-19 pandemic is a multifaceted stressor. Its impact suggests long-term psychological effects. Self-determination promotes flexibility of goals and actions and helps to overcome the difficulties caused by stress.

**Objective:**

To analyze coping strategies during the COVID-19 pandemic presented in Russian scientific studies (RQ1), and their relationship with self-determination (RQ2).

**Design:**

Relevant studies (2020-2022) were selected from the Russian citation index (RSCI) database. Strict selection criteria were used. Twenty-four articles were selected for the final review. For dynamic analysis, four stages of the pandemic were identified.

**Results:**

Prevailing coping strategies have changed over time. At the beginning of the pandemic, respondents used familiar coping mechanisms. Six months later, active coping strategies were more often used, but deprivation and avoidance strategies increased. A year later, there was an increase in denial and avoidance strategies. Using non-constructive coping strategies may indicate that, due to the long course of the pandemic, meeting basic psychological needs became increasingly frustrated, leading to helplessness, alienation, and lack of control. Later dynamics reflect the growth of effective coping strategies and confirm that when basic needs are blocked for a long time, people seek alternative ways to satisfy them.

**Conclusion:**

The dynamics of coping strategies during the pandemic reflected their close relationship with basic psychological needs, as described in the theory of self-determination. The results confirmed the importance of self-determination as a dispositional variable in predicting coping mechanisms.

## Introduction

In psychology, a pandemic is viewed as a multi-aspect stress factor, with hard-to-predict and far-reaching consequences for mental and physical health. Specialists evaluate the current situation around the COVID-19 pandemic as psycho-traumatizing, with such distinct characteristics as unpredictability and uncertainty, leading to elevated social and psychological risks (Bojko et al., 2020; Epishin et al., 2020; Grishina & Lupulyak, 2020; Malyh & Sitnikova, 2021; Pervushina & Shabalin, 2020).

A meta-analysis of studies published in 2020-2021 in the WSCC database showed that at the beginning of the pandemic in different countries, people were confused at the sudden onset of stress, unprecedented social restrictions, loss of a sense of security and stability (Kostromina et al., 2022). Many studies have described primarily emotional problems: symptoms of depression (27.5%), anxiety (26.9%), distress (26.5%) (Li, 2020; Sani et al., 2020), fear of COVID-19 infection and probability of death (Pakpour & Griffiths, 2020; Rasskazova & Tkhostov, 2021; Ravens-Sieberer et al., 2021), and such social problems as concern for the health of loved ones, uncertainty about fulfilling commitments (e.g., school, finances, work), difficulty adapting to remote work (Epishin et al., 2020; Kozhina & Vinokurov, 2020; Poluekhtova et al., 2020; Toscano et al., 2022), financial uncertainty (Cao et al., 2020; Islam et al., 2020; Khan et al., 2020; Sundarasen et al., 2020).

According to other studies, social restriction resulted in the following experiences: deprivation, dissatisfaction of needs , and low tolerance for uncertainty (Amin, 2020; Ausin et al., 2020; Brooks et al., 2020; Moore et al., 2020; Satici et al., 2020; Sood, 2020). A recent meta-analysis that included 123 systematic reviews of the prevalence of depression, anxiety, and posttraumatic stress disorder (PTSD) during the pandemic in different countries (December 2019 to August 2022; Russia was not included) showed heterogeneous data across populations. A slight but consistent deterioration of mental health was found at the beginning of the pandemic and during social restrictions in the general population and in people with chronic somatic diseases. Symptoms of depression increased during periods of social restriction, while signs of anxiety did not (Witteveen et al., 2023).

Many studies have focused on ways of coping with the pandemic. For example, a study of high-performance athletes during self-isolation and periods of uncertainty about competitions found that the athletes’ use of cognitive restructuring and emotional calming was significantly negatively correlated with negative emotional states such as depression, stress, anxiety, and fatigue. The reason maybe that high-performing professional athletes are more experienced in coping with competition-related anxiety (Leguizamo et al., 2021). Other factors have had an important role in the occurrence of negative emotional states, including gender, type of sport, qualifications, nationality, and personality traits. The role of adaptive and maladaptive perfectionism in relation to dominant mental states during social isolation and the choice of preferred coping strategies has been shown (lancheva, et al., 2020). A meta-review of articles with samples of healthcare workers and employees of other professions found that levels of psychological distress (stress or emotional burnout) differ in different countries. An important factor in reducing stress to a minimum was job involvement, such as helping employees to understand their contribution to organizational goals and their own personal growth (Adanaque-Bravo et al., 2023). In another metasurvey, individual and group psychological strategies, family support, and professional training were the most frequently cited coping strategies for healthcare workers (Chutiyami et al., 2022).

In countries with different cultures and economic structures, people are simultaneously faced with uncertainty, unpredictability, novelty, the impossibility of realizing their aspirations, and everyday restrictions. Studies show that at the heart of social disadvantage is a blockage of fundamental psychological needs. As a stressful event, the pandemic has elicited a specific response: a set of actions to overcome conflict, ensure safety, and maintain a sense of “normality” in life.

Relying on Western studies (Kostromina et al., 2021; Ntoumanis et al., 2009), we suggest that self-determination has an important role in overcoming the negative psychological consequences of the pandemic, acting as an important personal resource for overcoming stress and reducing its negative impact (Bakker et al., 2021; Ntoumanis et al., 2009). Self-determination is a set of characteristics that provide free and autonomous regulation of ones life. According to the self-determination theory (SDT) of E. Desi and R. Ryan (Ryan & Deci, 2000), the need for autonomy, competence, and relatedness are critical variables in behavioral regulation and psychological well-being. Self-determination requires a deep level of self-consciousness and makes for flexible aims and actions under stress (Amiot et al., 2008). Strategies of self-determination, such as helping those in need, searching for resources, taking initiative, and clear and transparent planning, help to overcome difficulties in the professional sphere caused by the COVID-19 pandemic (Klimochkina et al., 2022; Zinchenko et al., 2020; Zinovyeva et al., 2021).

This study conducted a meta-analysis of Russian studies during the pandemic to identify the main strategies for coping with the psychological threats of the pandemic and to analyze them in terms of self-determination and the realization of needs.

The aim of the study was to analyze strategies of coping with the experiences caused by the COVID-19 pandemic (2020-2022) as presented in Russian-language journals. We studied ways of coping with specific stressful experiences that emerged in the population during the pandemic and their dynamics over time.

## Methods

A search of full-text publications in the RSCI database (Russian citation index) was performed on July 11, 2022. The search was limited to peer-reviewed articles of all types, published from January 1,2020 to June 30,2022.

In order to identify all possible publications relevant to the research topic, the query was for “All fields” by two groups of keywords, with the AND operator between them. The following steps represented the search algorithm:

1. First search line: All fields — Coping OR coping strategies OR coping behavior;
2. Second search line: All fields — Covid OR coronavirus infection OR coronavirus.

The exclusion criteria were: theoretical reviews, abstracts without study descriptions, articles without empirical data, without a focus on coping strategies, or those that described unique samples.

A total of 102 results were obtained. Theoretical reviews (10 articles), abstracts without study descriptions (5 articles), and duplicate articles (1 article) were then excluded, leaving 85 articles for further analysis. All of these articles were reviewed for relevance to the review’s aim. Two reviewers checked them for inclusion or exclusion from the primary analysis according to the defined criteria.

**Figure 1.**
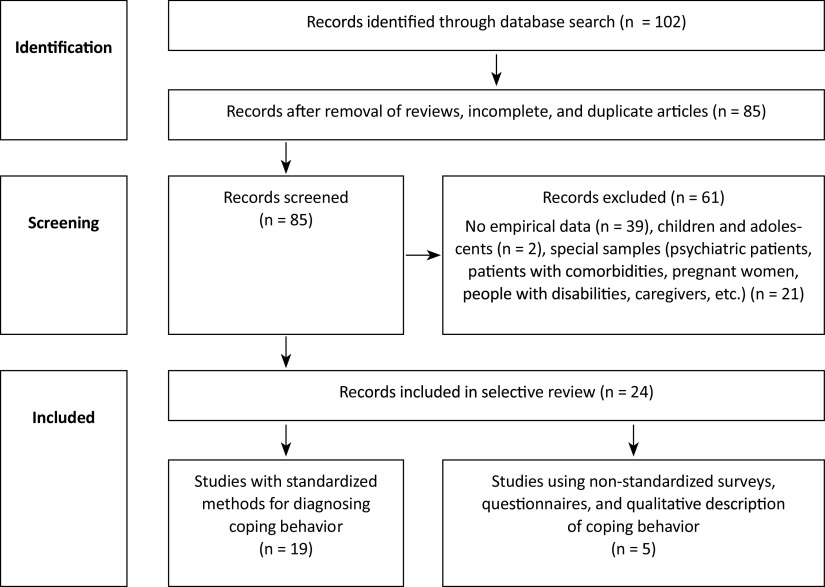
PRISMA flowchart of systematic literature review process

Furthermore, 61 articles that did not contain empirical data, did not focus on psychological coping, or described unique samples (pregnant women, people with disabilities) or a specific age group (children or the elderly) were excluded. The final sample included 24 publications whose full texts were suitable for the review analysis and were comprehensively studied. The selection process of articles is shown in the PRISMA flow chart in *Figure 1*.

### General Characteristics of the Selected Publications

Studies conducted during the pandemics two and a half years (2020, 2021, and the first half of 2022) were analyzed. Above all, we were interested in the variety of ways in which people overcome these specific experiences and how these change over time.

For analysis, the studies were divided according to the predominant methods for studying coping behavior (see *Table 1*):

1. Standardized methods for diagnosing coping strategies according to the theoretical framework;
2. Non-standardized methods, including surveys, questionnaires, and qualitative descriptions of coping behavior. Methods that had not been tested in Russia were included and interpreted as qualitative.

**Table 1:** Distribution of articles selected for review by type of study methods

Type of Study Sample Size (N) Time	Sample Size (N)	Time of the survey	Relevant Studies
Studies with standardized methods for diagnosing coping behavior			
Brief COPE instrument (Carver, 1997), including national versions and selected subscales	558	Spring 2020	Kryukova, 2020
	310	Spring 2020	Opekina, 2020
	232	Spring 2020	Bojko, 2020
	70	Spring 2020	Sergeeva,2021
	1140	Spring 2021	Shpakov, 2021
Ways of Coping Questionnaire (Lazarus & Folkman, 1988), including national versions and selected subscales	218	Spring 2020	Kislyakov, 2020
	248	Spring 2020	Kuftyak, 2020
	119	Spring 2020	Shishkov, 2021
	629	Spring 2020	Korotkova, 2021
	169	Spring 2020	Pogonysheva, 2022
	64	Spring 2020	Kora, 2022
	736	Fall 2020	Kameristaya, 2021
	186	Spring 2021	Kuvaeva, 2021
	70	Fall 2021	Ochirova, 2022
	67	Fall 2021	Mamina, 2022
Other standardized methods for diagnosing coping strategies	55	Spring 2020	Yarmysh, 2020
	102	Fall 2020	Kozlova, 2020
	209	Fall 2020	Kovaleva, 2021
Studies using non-standardized methods, questionnaires, of coping behavior and qualitative description			
Foreign methods that are currently in process of approbation in Russia	248	Spring 2020	Govorkova, 2020
Studies based primarily on qualitative research methods	279	Spring 2020	Prilutskaya, 2020
	306	Spring 2020	Frolova, 2021
	14	Spring 2020	Petrakova, 2021
	136	Spring 2020	Volodina, 2022

The results were rated according to the time of the survey and corresponded to one of the periods described in *Table 2*.

**Table 2:** Number of articles selected for review depending on the time period of the survey

Time period	Total number in this period of surveys	Total sample size
Spring 2020	16	3486
Fall 2020	3	1047
Spring 2021	2	1326
Fall 2021	2	137

The largest number of Russian studies concerned the beginning of the pandemic; after that, there has been an apparent decrease in studies of coping strategies during the pandemic. This maybe due to the gradual adaptation of society to the pandemic, a change in the main topics of research interest towards private issues not involving coping mechanisms, and, ultimately, a decline in interest in the topic of the pandemic as a whole.

Researchers’ data on coping behavior during the COVID-19 pandemic were analyzed in chronological order.

## Results

### Coping Strategies at the Beginning of the Pandemic (Spring 2020)

This period saw the largest number of Russian-language publications. We grouped them according to the methodological apparatus used by the authors. The studies using the following methods are presented below: Lazarus’ Ways of Coping Questionnaire (WCQ; these studies were the most numerous), the COPE methodology, the Proactive Coping Inventory, the Coping Inventory for Stressful Situations, and the Coping Flexibility Scale. Finally, studies using non-standardized methods are presented.

### *Analysis of Data on Coping Strategies Obtained with the* WCQ

Among the standardized methods, the Ways of Coping Questionnaire in various Russian-language adaptations was the most frequently used, covering a total sample of 1,447 people.

A uniform quantitative analysis of the results of studies using the WCQ is rather complicated, since different data presentation formats were used. Some authors give the percentages of occurrence of each type of coping, while others present only the most popular coping strategies or average data for each of the coping mechanisms. Thus, each study using this questionnaire became practically unique in content. Therefore, the description of results below further summarizes and analyzes the prevalence of different coping strategies, coping mechanisms, and associated factors.

Most of the studies noted that in the first months of the pandemic, some of the most frequently encountered coping strategies were avoidance (Kislyakov, 2020; Kora, 2022; Korotkova et al., 2021; Pogonysheva & Protasova, 2022) and distancing (Kislyakov, 2020; Kora, 2022; Pogonysheva & Protasova, 2022). Thus, the primary reaction to stress was accompanied mainly by manifestations opposite to self-determination. But several works (Kislyakov, 2020; Shishkov et al., 2021) also indicated a high frequency of such coping strategies as “decision planning” and “search for social support” (Kislyakov, 2020; Korotkova et al., 2021; Pogonysheva & Protasova, 2022; Shishkov et al., 2021). These results were obtained in studies where the predominant sample was younger people (students and young adults). In general, it can be noted that multidirectional results may indicate a variety and mixed repertoire of coping strategies in the initial stage of the pandemic.

Several papers described the interrelation of the preferred coping strategies with life features before the pandemic. For example, Shishkov et al. (2021) found that people living alone most often used the strategies of “planning” (35.3%), “seeking social support” (33.6%), and “accepting responsibility” (27.7%), which are characteristic of self-determination. The authors attributed this to the fact that those living alone were used to planning their own time, and the onset of the pandemic had less of an impact on their daily routines and ability to plan their time. Respondents demonstrated similar results in objective isolation (associated with living alone, away from loved ones) or subjective isolation (related to the goal of shielding oneself or others from contact).

A relationship between stress level and preferred coping strategies was also found (Kuftyak & Bekhter, 2020). Participants with a low stress level more frequently chose proactive coping (p = 0.0001), and those with a high stress level searched for instrumental support (p = 0.04). Thus, the level of stress is associated with the ability to implement proactive coping strategies.

### Analysis of Data on Coping Strategies Obtained with COPE

Using the COPE questionnaire (total sample of 1,170 people) showed great similarity of the results obtained by different authors (Bojko et al., 2020; Kryukova et al., 2020; Opekina & Shipova, 2020; Sergeeva & Kubekova, 2021). Generally, the prevalence of such strategies as acceptance, positive overestimation, emotional support, and active coping was noted. The most rarely encountered strategies were denial, avoidance, and substance use. It should be noted that the data on many coping strategies is close to that empirically deduced in the pre-pandemic norms. Differences are observed only for some nonconstructive strategies: denial, substance use, and self-blaming (their normative index is higher than that obtained in the present research).

Comparison of students who had and did not have COVID-19 (Shpakov et al., 2021) showed significant differences in their use of individual coping strategies: students who did not have COVID-19 more often used acceptance strategies (5.8 ± 1.44 and 5.5 ± 1.44, respectively, p = 00.1) and planning strategy (6.5 ± 1.33 and 6.2 ± 1.36, respectively, p = 0.05). The strategy of turning to religion was more common among students who recovered from Covid (3.7 ± 1.81 among healthy students, 4.0 ±1.91 among recovered patients, *p =* 0.05). Analysis of Data on Coping Strategies Obtained with Other Standardized Methods

In addition to the preferred WCQ and COPE methods, researchers used other questionnaires to assess coping behavior. The Proactive Coping Inventory questionnaire (Greenglas et al., 1999), showed (Kuftyak & Bekhter, 2020) that the preferred types of proactive coping were:

- Proactive coping (a persons attitude toward a problematic situation as a source of positive experience) (17.2%);
- Reflexive coping (representation of possible behavioral options, cognitive evaluation of resources, and prediction of outcomes) (14.3%); and
- Preventive coping (ability to anticipate difficult situations by relying on experience) (13.75%).

The least preferred coping method was strategic planning (the ability to plan future actions with differentiation of individual tasks) (7.55%). The authors found that coping strategies are related to the level of stress and the reaction to the stressful event. Thus, respondents with a low level of stress significantly more often used methods of proactive coping (18.3% in the group with a low level of stress, 16.1% in the group with a high level of stress, *p =* 0.0001). Respondents with high levels of stress significantly more often used instrumental support (9.6% in the low-stress group, 10.6% in the high-stress group, *p =* 0.04). The authors note that prolonged mental strain caused by self-isolation reduces a persons ability to assess their resources and positively evaluate a stressful situation.

One of the studies (Yarmysh, 2020) provides data obtained using the Coping Inventory for Stressful Situations questionnaire (Endler & Parker, 1990). It showed that the predominant coping mechanisms in the COVID-19 pandemic are relatively adaptive ones. Cognitive coping mechanisms are the most popular (72%), and emotional coping mechanisms are the least popular (21%). The authors note that the identified coping mechanisms help to cope with difficulties, but only in situations with little stress and that are not very significant for the individual.

Another study (Govorkova et al., 2020) used the Coping Flexibility Scale (Gembeck & Skinner, 2018), which allows for assessing the degree of flexibility and stability of the coping system. The study showed that the frequency of different types of coping flexibility does not differ from the normal distribution. At the same time, the authors note that the flexible type of coping reduces stress level.

### Analysis of Data on Coping Strategies Obtained with Non-Standardized Methods

Four studies used qualitative methods, including authors’ questionnaires (Frolova & Vysockaya, 2021; Prilutskaya et al., 2020; Volodina et al., 2022) and semi-structured interviews (Petrakova et al., 2021), with a total of 735 people.

The authors distinguish cognitive, emotional, and behavioral ways of coping with stress in a situation of self-isolation in connection with the pandemic. Cognitive ways include a search for new ideas, the ability to find meaning and positive aspects in the current situation, the actualization of their creative skills, the reflection of experiences and personal qualities, and the desire to understand other people (Frolova & Vysockaya, 2021; Petrakova et al., 2021), as well as self-organization and the selection of new priorities (Volodina et al., 2022). Emotional ways of coping with forced self-isolation are represented by the generation of new positively colored emotions in unfamiliar conditions, joy from the opportunity to do something that has long been planned, self-support of ones sense of humor, empathy with other people, and a feeling of unity with the whole world (Frolova & Vysockaya, 2021). Behavioral ways of coping include communication with friends, family, and classmates (Petrakova et al., 2021; Volodina et al., 2022), sports and hobbies (Petrakova et al., 2021; Prilutskaya et al., 2020), video games, self-education, and a focus on professional (clinical) activities (Prilutskaya et al., 2020).

It has been shown that girls, in comparison with boys, have a more pronounced resource of communication; however, such resources as self-organization, volitional qualities, and self-motivation are similar (Volodina et al., 2022).

### Coping Strategies Six Months After the Start of the Pandemic (Fall 2020)

There were considerably fewer studies conducted in the fall of 2020. We found three studies devoted to the chosen topic. The focus of such research had shifted from direct study of prevailing coping strategies to their connection with the level of stress and the intensity of the personal situation.

It was shown that six months after the beginning of the epidemic, young people more often used active coping strategies (reflexive and preventive coping, planning). Young people more often used strategies of seeking support, positive reassessment, confrontation, self-blaming, fantasizing, distancing, and avoidance. In adults, future orientation, predicting the situation, and planning actions based on available resources prevailed (Kameristaya, 2021).

Based on the data from the Coping Behavior in Stressful Situations questionnaire, it was shown that the most frequently used coping strategies were problem-solving, avoidance, and focusing on emotions (Kovaleva et al., 2021; Kozlova & Kostrigina, 2020). However, the authors note that these data hardly differ from normative parameters, which allows us to conclude that at the time of the survey, most of the respondents were in the phase of resistance to the stressful situation.

The study, which included four consecutive measurements from September to December 2020 showed that the number of respondents with a high degree of coping strategies focused on emotion and avoidance increased simultaneously with an increase in the use of distraction and social distraction strategies. According to the authors, this maybe a sign of depletion of the individuals adaptive resources (Kovaleva et al., 2021).

Finally, it was suggested that the preferred type of proactive coping maybe related to the subjectively perceived level of tension in the situation (Kameristaya, 2021). This allows us to assume a growth of self-determination in prolonged stress during COVID-19.

### Coping Strategies One Year After the Start of the Pandemic (Spring 2020)

We found only two studies analyzing preferred coping strategies a year after the pandemic's beginning (Kuvaeva & Strel'nikova, 2021; Shpakov et al., 2021), indicating a steady decline in interest in the topic.

In the first study, “Coping Behavior in Stress Situations” by N.S. Endler and J.A. Parker, the questionnaire of coping methods by R. Lazarus and S. Folkman was used (Kuvaeva & Strel'nikova, 2021). It was shown that while coping with the pandemic, respondents most frequently used positive reassessment strategies (11.33 ± 3.89), self-control (9.05 ±3.42), problem-solving planning (8.65 ±3.04), and distancing (8.23 ± 2.83), which corresponded to the respondents’ stable coping styles. Furthermore, the authors concluded that respondents were more likely to turn to problem-solving-oriented coping (57.33 ± 10.00) during this period in stressful situations. On the other hand, respondents much less frequently used emotionally oriented (44.41 ± 12.69) and avoidant (41.94 ± 11.05) coping types. The persistent avoidance style was primarily manifested in visiting stores and restaurants, a tendency to sleep for a long time, overeating, watching TV, etc.

Another study was conducted by a team of authors using the COPE technique on an impressive sample of 1,140 people (Shpakov et al., 2021). It was shown that the preferred coping strategies were planning, active coping, positive reformulation, and personal growth, acceptance, seeking instrumental social support. The data presented by the authors allowed us to compare the scores on this methodology in 2020 and 2021 and to identify differences, which will be analyzed below.

Comparative studies about peoples experiences during the pandemic became possible during this period. Thus, a comparison of preferred coping strategies among respondents infected and non-infected by COVID-19 was conducted (Kuvaeva & Strel'nikova, 2021). It was shown that the respondents who had contracted the coronavirus infection demonstrated increased social activity: they tried to be out in public, visited, spent time with a friend or loved one, and asked for advice from a significant other. According to the authors, the desire for social contact in stressful situations as a stable and habitual way to relieve stress may have contributed to COVID-19 infection and the spread of the disease. However, the study of interrelations between stable coping styles and strategies revealed a more complex structure in the group of respondents who had Covid-19.. Stress avoidance is associated with a more active search for social support and self-blaming: the more they immerse themselves in their experiences, the less they think about problem-solving. They are less likely to switch to other activities.

### Coping Strategies at the End of the Pandemic (Fall 2021)

We found two studies (Mamina et al., 2022; Ochirova & Chuvasheva, 2022) devoted to the analysis of coping strategies at the end of the pandemic. The Coping Behaviors questionnaire by Lazarus was used in both studies. Both studies focused not on the coping strategies themselves, but on their interrelation with the level of stress and anxiety experiences of the individual.

Mamina et al. (2022) showed that the choice of coping strategy was connected with the persons level of anxiety. Thus, among students with a high level of anxiety, the most frequent coping strategies were avoidance (67.1 ±0.13) and distancing (65.8 ±0.27). The authors note that using intellectual techniques characterizes distancing, for example, rationalization, shifting attention, detachment, humor etc. The avoidance strategy is characterized by denial of the problem, fantasizing, etc. Thus, non-adaptive coping is more typical for students with high situational anxiety.

Such coping strategies as distancing (61.6 ±0.11) and solution planning (54.9 ± 0.26) were most common among students with an average level of anxiety. This group of students is characterized by a combination of constructive and nonconstructive coping. Most researchers consider decision planning to be an adaptive strategy, as it promotes constructive resolution of difficulties. This group of students is more characterized by the mixed type of coping.

Finally, among students with a low level of anxiety, the strategies of solution planning (60.8 ±0.19) and positive reevaluation (58.6 ±0.32) are most expressed. These ways of coping with stress are fully adaptive.

Similar results were obtained by Ochirova and Chuvasheva (2022), who found that the most frequent coping strategies were avoidance, distancing, confrontation, and positive reassessment. However, the authors show that the preferred coping strategy is connected with the stress level and actual experiences. The higher the stress level of the subjects, the more they are inclined to use the strategy of avoidance; at a low level of stress, the participants choose the strategy of positive reassessment.

## Discussion

Analysis of the Russian-language publications on coping strategies during COVID-19 demonstrates unstable interest. Most studies occurred at the beginning of the pandemic, and then their number decreased significantly. Studies at the end of the pandemic are characterized by location-based studies and small sample sizes. In contrast, a review of foreign studies showed a steady increase in surveys during the two years of the pandemic, some of which focused on the dynamics of coping strategies and others on their comparative analysis by age, country, and living conditions (Kostromina et al., 2022).

The Russian comparison studies mainly focused on predominant coping strategies and whether they differed from pre-pandemic distribution. In the late period, the researchers’ main focus shifted from the direct study of predominant coping strategies to their connection to stress and personal tension; comparative studies of coping strategies were carried out in groups with different stress levels. A year after the pandemic’s beginning, the researchers’ main interest was in comparing predominant coping strategies between those infected with COVID-19 and those not.

A comparison of Russian-language publications is difficult in part because of the lack of a common methodology in the study of predominant coping strategies, the lack of uniformity in methods and in processing of results. Nevertheless, it is possible to get a general idea of how the repertoire of coping strategies has changed during the pandemic in Russia. At the pandemic’s beginning, adaptive and relatively adaptive defense mechanisms were used a great deal. Some researchers note the predominance of non-constructive, escape-oriented coping strategies and a variety of coping strategies in general. It is worth noting that different coping strategies’ distribution frequency is the same as the normative and reiterates pre-pandemic norms. Foreign studies’ analysis yielded similar results, demonstrating a variety of coping strategies at the pandemics beginning (Maykrantz et al., 2021; Morales-Rodriguez, 2021; Park et al., 2021).

With adaptation to the situation, there was a selection of effective coping strategies. Some of them were weakened, such as cooperative family activities or virtual communication (Adams & Smith, 2021). Others increased (e.g., active behavior strategies, seeking social support, or reframing) (Awoke et al., 2021; Babicka-Wirkus et al., 2021; Kryukova et al., 2021). In Russia, the dynamics were characterized by two trends. On the one hand, active coping strategies such as planning, reflexive, and preventive coping have increased. On the other, there was a slight increase in deprivation and avoidance strategies.

The worldwide trend of a gradual increase in proactive coping strategies during adaptation to COVID-19 (Diaz et al., 2021) further confirms that when basic psychological needs (autonomy, competence, and relatedness) have been blocked for a long time, people start looking for an outlet to satisfy them and choose those coping strategies that can do so (Ntoumanis et al., 2009). During the pandemic, social networking, peer support, teamwork, self-confidence, problem-solving, and self-care were the most frequently used coping strategies (Finstad et.al., 2021). This confirms the idea that in situations of isolation, preference is given to those coping strategies that directly or indirectly satisfy the need for self-determination. Overall, researchers note the effectiveness of proactive coping strategies among respondents with low stress and anxiety levels (Kameristaya, 2021; Kuftyak & Bekhter, 2020; Mamina et al., 2022; Ochirova &: Chuvasheva, 2022). This result confirms prior observations that proactive coping relates to readiness to act purposefully (Schwarzer & Taubert, 2002). However, targeted action is difficult when stress levels are high, so focusing on the problem helps to reduce anxiety and depression (Finstad et.al., 2021). Perhaps this circumstance can explain the fact that after one year of the pandemic, there has been an increase in denial strategies (e.g., reluctance to acknowledge the existence or threat posed by COVID-19) and avoidance strategies (e.g., avoidance of discussing the danger posed by COVID-19) among Russians. Similar trends have been reported in foreign studies. In the early phases of the pandemic, avoidance strategies allowed workers to limit their sense of helplessness and incompetence, contributing to resilience (Maiorano et al., 2020). In later periods of the pandemic, this may be due to general societal fatigue with the situation.

In general, the review of both Russian and foreign studies shows that in a situation of coping with anxiety and stress, any of the coping strategies is somehow connected with the realization of the needs for self-determination (Kostromina et al., 2022). The use of emotional coping strategies is largely determined by the need to regulate the self in response to stressors assessed as threats (Amiot et al., 2008). These strategies focus on self-management through physical activity, meditative practices (Burch et al., 2021), and the release of emotions (Zimmer & Dunn, 2021). Coping with helping others, learning new activities, and organizing and communicating in the pandemic situation allowed for a sense of connection with others, “distraction from stress,” self-development, and increasing one’s own competence. Coping focused on situational awareness and reframing supported the need for autonomy by rethinking and giving meaning to what was happening. Such coping is recognized as one of the most successful means of promoting well-being (Kashdan & Rottenberg, 2010) in stressful and crisis situations.

## Conclusion

The trends in the dynamics of coping strategies during the pandemic reflect their close relationship with basic psychological needs in the theory of personality self-determination. This review showed a transition from confusion and habitual, pre-pandemic coping strategies to more effective ones, confirming that when basic needs are blocked for a long time, people seek a way to satisfy them. A gradual increase in active coping is an actualization of the need for competence, realized in conditions of social restrictions. An increase in coping associated with the search for new activities and hobbies helps satisfy the basic need for connectedness. Positive thinking and reframing strategies allow one to shift the locus of control from external circumstances to internal ones, to feel ones own role, thereby supporting the need for autonomy and competence, while setting clear goals and planning activity satisfies all three basic needs. The results of the studies reviewed confirmed the importance of self-determination as a dispositional variable in predicting patterns of coping with stress.

## Limitations

As noted, most empirical publications could not be included in the final analysis because they needed to provide more empirical evidence. Difficulties in the analysis were also associated with the use of different variants of the Russian adaptation of the applied methods, as well as the presentation of results in different formats.
